# Validity and Reliability of the Basic Psychological Need Satisfaction and Frustration Scale Among Cancer Survivors in Korean Healthcare Contexts

**DOI:** 10.3390/healthcare12242535

**Published:** 2024-12-16

**Authors:** Hyun-E Yeom, Jungmin Lee

**Affiliations:** 1 Department of Nursing, Chungnam National University, Munhwaro 266, Junggu, Daejeon 35015, Republic of Korea; 2 Korea Research Institute for Vocational Education & Training, Social Policy Building, Sejong National Research Complex, 370 Sicheong-daero, Sejong-si 30147, Republic of Korea

**Keywords:** patient self-determination act, motivation, needs assessment, validation study, psychometric, cancer survivors

## Abstract

Background/Objectives: Basic psychological needs are essential for fostering motivation, self-regulated behaviors, and overall well-being. For cancer survivors, fulfilling these needs is crucial for coping with the various challenges of survivorship and for enhancing psychosocial health. This study aimed to assess the validity and reliability of the Korean version of the Basic Psychological Needs Satisfaction and Frustration Scale (K-BPNSFS) in a cancer survivor population. Methods: A cross-sectional design was employed, involving 367 community-dwelling cancer survivors. Construct validity was assessed using confirmatory factor analysis with multiple fit indices, while convergent validity was examined through Pearson’s correlation coefficients. Reliability was evaluated using internal consistency, inter-item correlations, and item-total correlations. Results: The findings confirmed a robust six-factor structure of the K-BPNSFS, which includes satisfaction and frustration dimensions corresponding to autonomy, relatedness, and competence needs. Convergent validity was supported by significant correlations with relevant constructs, aligning with the scale’s theoretical underpinnings. Reliability analysis demonstrated high internal consistency across all dimensions, with strong Cronbach’s alpha values and substantial item-total and inter-item correlations. Conclusions: This study establishes the K-BPNSFS as a valid, reliable, and culturally relevant instrument for assessing the basic psychological needs of Korean cancer survivors. Application of this scale provides critical insights into the unique psychological needs of this population, supporting the development of targeted healthcare strategies to enhance intrinsic motivation, self-care, and overall quality of life.

## 1. Introduction

With advances in medical techniques improving survival rates, cancer is now recognized as a chronic condition requiring long-term management throughout the survivorship journey [1,2]. Cancer survivors encounter multifaceted physical and psychological challenges during the phases of active therapy, recovery, and follow-up, which significantly affect their fundamental health needs [2]. An expanding body of health literature highlights the strong association between individuals’ psychological needs, intrinsic motivation, and their capacity to navigate life’s complexities, underscoring the importance of addressing these diverse needs [3]. Empirical research further demonstrates the pivotal role of psychological needs in enhancing health outcomes for cancer survivors [4,5,6,7]. These needs have been linked to various aspects of well-being, including physical and mental health for testicular cancer survivors [4], social well-being in older cancer survivors [5], engagement in physical activity among colorectal cancer survivors [6], and self-management in breast cancer survivors [7]. In light of the enduring challenges of survivorship, it is essential to understand the psychological needs of cancer survivors to enhance motivation and behaviors that lead to an overall improved quality of life.

Self-determination theory (SDT), a conceptual framework guiding psycho-cognitive behavioral studies, focuses on three basic psychological needs (BPN)—autonomy, relatedness, and competence—that are innate traits facilitating motivation, leading to behavioral changes and personal growth [8,9,10]. Autonomy refers to the experience of making choices and acting according to one’s own will, giving the individual a sense of control over their actions and life [8]. Relatedness involves forming meaningful relationships with others, feeling connected and belonging, and experiencing support, understanding, and respect through social interactions [8]. Competence refers to the perception of effectively and successfully interacting with the environment, solving challenging tasks, and experiencing a sense of achievement by applying one’s abilities [8].

SDT posits that whether these needs are fulfilled or frustrated affects intrinsic motivation and self-driven behaviors that are linked to psychological well-being [9,11]. Aligned with this proposition, numerous empirical studies have demonstrated that satisfaction and frustration with the BPN are associated with distinctive aspects of self-regulated behaviors, psychological integration, and overall well-being in school education [12], sports [13], parenting [14], and healthcare [15]. Further, SDT postulates that the satisfaction and frustration of BPN are not reverse concepts [9,15,16], implying that the satisfaction and frustration of the BPN to be considered as independent features representing the unique status of BPN [9]. For instance, the healthcare literature emphasizes that the satisfaction of BPN is essential for fostering active engagement in healthy behaviors, such as maintaining regular physical activity and adhering to a balanced diet [7,17]. Conversely, the frustration of these needs is associated with maladaptive health behaviors, such as reduced intrinsic motivation for exercise [18] and adverse health outcomes [19]. These findings highlight the conceptual distinction between need satisfaction and frustration, demonstrating that they are not merely opposite ends of a continuum. This distinction underscores the importance of examining both aspects of BPN to fully understand the experiences of cancer survivors.

The Basic Psychological Need Satisfaction and Frustration Scale (BPNSFS) has been used as a valid tool for assessing the extent to which individuals’ BPN are fulfilled or thwarted [9,15,16]. Satisfaction with these needs represents feeling in control of one’s actions (autonomy), feeling connected to others (relatedness), and feeling competent (competence). In comparison, frustration reflects the status of these needs being unmet, such as feeling controlled or pressured (lack of autonomy), feeling isolated or socially disconnected (lack of relatedness), and feeling inadequate or ineffective (lack of competence). As such, the BPNSFS provides a comprehensive framework for assessing the satisfaction and frustration of core psychological needs.

The original BPNSFS has been translated into several languages, including Japanese [20], Italian [21], German [22], Polish [23], and Arabic [24]. Validation studies of these translations consistently confirm a six-dimensional structure that captures both satisfaction and frustration related to the three psychological needs (i.e., autonomy, relatedness, and competence). This dual focus on fulfilled and unmet needs offers a more nuanced understanding of psychological needs, enhancing its utility in health research and psychological interventions for individuals confronting health-related challenges. However, existing studies of medical contexts, including cancer survivors [4,5,6,7,8], have predominantly focused on the satisfaction of BPN, often overlooking the equally critical aspect of need frustration. Furthermore, while the BPNSFS has been validated for assessing BPN in individuals with specific health conditions, such as mental well-being/ill-being [23], depression [24], and HIV [25], its application to cancer survivors remains largely unexplored. This gap underscores the need for further research to evaluate its relevance and validity in this population.

Cancer survivors encounter numerous hardships, including distress from uncertainty, anxiety about recurrence, social isolation during treatment, and physical discomfort [1,2,4,5]. These challenges can profoundly impact their BPN. While BPN are regarded as a universal innate trait across diverse ages and socio-cultural contexts [8,9], limited research exists on the distinctive patterns of BPN satisfaction and frustration among cancer survivors. Although one study examined the BPN of cancer survivors in Korea [17], it concentrated exclusively on satisfaction, leaving the aspect of frustration unexamined. Utilizing the BPNSFS to this population offers an opportunity to provide health professionals with essential insights into the balance between positive and negative psychological experiences throughout the cancer survivorship journey. Considering the specific psychological and behavioral challenges faced by cancer survivors, this study aimed to investigate the validity and reliability of the BPNSFS within the Korean context. By establishing a critical foundation for understanding the psychological needs of cancer survivors, this research provides valuable insights to guide clinical practices aimed at enhancing their motivation, self-care behaviors, and overall quality of life.

## 2. Materials and Methods

### 2.1. Design

This cross-sectional study employed a methodological approach, utilizing secondary data from two primary studies. Study 1 explored the relationships among interpersonal, psychological, and behavioral characteristics and their impact on health-related quality of life in a sample of 220 breast cancer survivors. Study 2 investigated psycho-cognitive and social determinants of self-care behaviors in 147 blood-cancer survivors [26]. Both studies focused on understanding how psycho-cognitive traits influence active self-care and enhance overall well-being throughout the cancer survivorship journey.

### 2.2. Sample and Process

Participants were recruited through convenience sampling from outpatient and inpatient facilities at Chungnam National University Hospital in Daejeon, the fifth-largest city in South Korea. Eligibility criteria for both Study 1 and Study 2 included being 19 years or older and not currently undergoing active cancer-related treatment, such as surgery or hematopoietic stem cell transplantation. Participants were also required to have only primary breast or blood cancer, with no other cancer types present. Additionally, participants needed to exhibit healthy cognitive functioning, be capable of communicating and understanding the questionnaires, and to respond to each item. Exclusion criteria were established to remove individuals receiving palliative care at the end-of-life stage or those referred to hospice care.

This study utilized data from 367 participants, including 220 breast-cancer survivors and 147 blood-cancer survivors. In terms of statistical power, a sample size of 367 was sufficient to assess structural validity through confirmatory factor analysis (CFA), which requires at least a 10:1 ratio of cases to free parameters [27]. Given that the BPNSFS consists of 24 items, the necessary sample size should exceed 240 under these criteria. Thus, the sample size supported construct validity with adequate statistical power.

### 2.3. Measures

#### 2.3.1. Korean Version of BPNSFS

The BPNSFS had not previously been translated or validated in Korean. Therefore, the original English version of the BPNSFS [16] was translated into Korean following a rigorous five-step process for cross-cultural adaptation of measurement tools [28]. This process included translation, synthesis, back-translation, content validity review, and pilot testing to ensure linguistic accuracy and cultural relevance.

The translation and synthesis were performed by three bilingual Korean-English translators to maintain linguistic accuracy while ensuring cultural relevance. A professional editor conducted a meticulous back-translation, carefully identifying and resolving discrepancies to preserve the original questionnaire’s integrity and eliminate potential back-translation errors. This step was critical in ensuring the Korean version accurately reflected the original contents while being understandable to Korean respondents.

Following this process, an expert panel comprising six Korean psychologists specializing in SDT-based research and three oncology clinical nurse specialists reviewed the content validity of the translated items. Using the content validity ratio (CVR) [29], the experts evaluated the appropriateness of terminology, accuracy of meaning, and socio-cultural relevance of each item. All items achieved a CVR of 0.556 or higher, indicating that the panel deemed most items essential. This high CVR score reflects strong agreement among the experts regarding the importance of the items for measuring the intended constructs.

Finally, a pilot test was conducted with ten cancer survivors to assess the clarity, comprehensibility, and cultural appropriateness of the translated items. Feedback from the pilot participants was utilized to refine the questionnaire further, ensuring that all items were understandable and allowed for accurate responses. Following this rigorous process, the Korean version of the BPNSFS (K-BPNSFS) was finalized. The scale comprises 24 items representing six dimensions: needs satisfaction of autonomy (items 1 through 4), relatedness (items 9 through 12), and competence (items 17 through 20) as well as needs frustration of autonomy (items 5 through 8), relatedness (items 13 through 16), and competence (items 21 through 24). Each dimension includes four items rated on a 5-point Likert scale, with responses ranging from 1 (“definitely no”) to 5 (“definitely yes”).

#### 2.3.2. Self-Acceptance and Depression: Validating Autonomy Needs

To assess the conceptual relevance of autonomy needs, the constructs of self-acceptance and depression were employed, reflecting established research connections between autonomy, internal empowerment, and psychological well-being in cancer populations [30,31]. Self-acceptance was assessed using the Self-Acceptance dimension of Ryff’s Psychological Well-being [32,33]. This eight-item scale is designed to assess an individual’s positive evaluation and acceptance of themselves and their past, capturing their acknowledgment and integration of personal strengths and weaknesses. The scale has been validated in both Western and Korean populations [32,33]. Participants rated their level of agreement on a 6-point Likert scale, with higher scores indicating a more positive self-attitude and a greater capacity to accept various aspects of oneself. The measure demonstrated strong internal consistency in Study 1, with a Cronbach’s alpha of 0.809.

Depression was evaluated using the Patient Health Questionnaire-9, a nine-item measure validated in Korea [34]. Participants rated the frequency of specific depressive symptoms over the past two weeks on a 4-point Likert scale. Higher scores indicate more depressive symptoms. This scale showed sufficient reliability, achieving a Cronbach’s alpha of 0.858 in Study 2.

#### 2.3.3. Family Interaction: Validating Relatedness Needs

The conceptual relevance of relatedness needs was explored by evaluating family interactions, which play a crucial role in fostering a sense of belonging and connection [35]. Family interaction was measured using the validated Family APGAR scale, which assesses core dimensions of family functioning, including adaptation, partnership, growth, affection, and resolve among family members [36,37]. This scale comprises five items rated on a three-point scale (0 = “hardly ever”; 1 = “sometimes”; 2 = “almost always”), with total scores ranging from 0 to 10. Higher scores indicate stronger family interaction. The scale demonstrated strong reliability, with Cronbach’s alphas of 0.894 in Study 1 and 0.881 in Study 2.

#### 2.3.4. Self-Efficacy and Uncertainty: Validating Competence Needs

The constructs of self-efficacy and uncertainty were selected to evaluate the conceptual relevance of competence needs, aligning with the literature on cancer-related coping and perceived competence [38,39]. Self-efficacy was measured using a 10-item General Self-Efficacy Scale, which has been validated across diverse populations [40]. Respondents rated their perceived competence in various situations on a 4-point Likert scale, with higher scores indicating greater self-efficacy. The scale demonstrated excellent internal consistency in Study 1, with a Cronbach’s alpha of 0.916.

Uncertainty was assessed using Mishel’s Uncertainty in Illness Scale, a validated instrument designed to measure psychological distress stemming from ambiguity and complexity in cancer populations, including Korean individuals [41,42]. The scale comprises 23 items, with responses rated on a 5-point Likert scale, where higher scores indicate greater levels of uncertainty regarding health-related concerns. In Study 2, the scale demonstrated good internal consistency, with a Cronbach’s alpha of 0.837.

#### 2.3.5. General and Health-Related Characteristics

Demographic information, including age, sex, educational level, marital status, living arrangements, employment status, and household income, was collected. Health-related characteristics were also documented, including general health status, body mass index, time since diagnosis, and cancer treatment history.

### 2.4. Ethical Consideration

The study protocols, objectives, and all associated methodologies for primary studies 1 and 2 were approved by the Institutional Review Boards at Chungnam National University (202101-SB-009-01) and Chungnam National University Hospital (2021-06-080-006), respectively. Data collection commenced after each participant provided written informed consent, in accordance with the ethical standards established by the institutional review boards. This process ensured compliance with the ethical guidelines for conducting research involving human subjects.

### 2.5. Statistical Analysis

This study conducted a series of evaluations to assess the construct validity of the K-BPNSFS, focusing on its six-factor structure and criterion-related validity. Data were analyzed using Mplus version 7.0 (Muthén & Muthén, Los Angeles, CA, USA). Preliminary and descriptive statistics for all study variables were computed to provide an overview.

To evaluate construct validity, the structural model of K-BPNSFS was examined using CFA with maximum likelihood estimation. Multiple indices were applied to assess the goodness of fit: χ^2^/df (<3), Comparative Fit Index (CFI > 0.90), Tucker–Lewis Index (TLI > 0.90), Root Mean Square Error of Approximation (RMSEA < 0.08), and Standardized Root Mean Square Residual (SRMR < 0.08) [43,44]. The Average Variance Extracted (AVE) was calculated from the CFA, with a cut-off value of 0.50 used to determine the adequacy of validity.

For criterion-related convergent validity, Pearson’s correlation coefficients between K-BPNSFS sub-dimensions and relevant variables were calculated: self-acceptance and depression for autonomy needs, family interaction for relatedness needs, and self-efficacy and uncertainty for competence needs.

Internal consistency was assessed using Cronbach’s alpha coefficients for the K-BPNSFS sub-dimensions, composite reliability (CR) derived from CFA, and item-total and inter-item correlations. A CR value above 0.70 and a Cronbach’s alpha of 0.60 or higher were considered indicators of acceptable reliability, indicating that the items measure a consistent underlying construct [45,46]. Additionally, item-total correlations (ranging from 0.30 to 0.80) and inter-item correlations (exceeding 0.15) were taken into consideration [47,48]. These thresholds were applied to ensure that the items contribute meaningfully to the overall scale and accurately capture the construct through its components. This process further confirms the scale’s reliability and validity in measuring the psychological construct of interest.

## 3. Results

### 3.1. Descriptive Characteristics of the Participants

The average age of 367 participants was 54.87 years (SD = 11.78). Most participants were married (79.8%) and had an educational background beyond high school graduation (81.5%). Overall, they reported moderate to good health status, with an average rating of 3.02 out of 5 (SD = 0.90) and a mean BMI of 23.22 (SD = 3.60). The average time since the initial cancer diagnosis was 25.41 months (SD = 22.16). Among the breast-cancer survivors, 188 out of 220 underwent lumpectomy or mastectomy. Among the blood-cancer survivors, 127 received only chemotherapy, while 53 underwent hematopoietic stem cell transplantation.

### 3.2. Confirmatory Factor Analysis for the Six-Factor Structural Model

**Figure 1:**
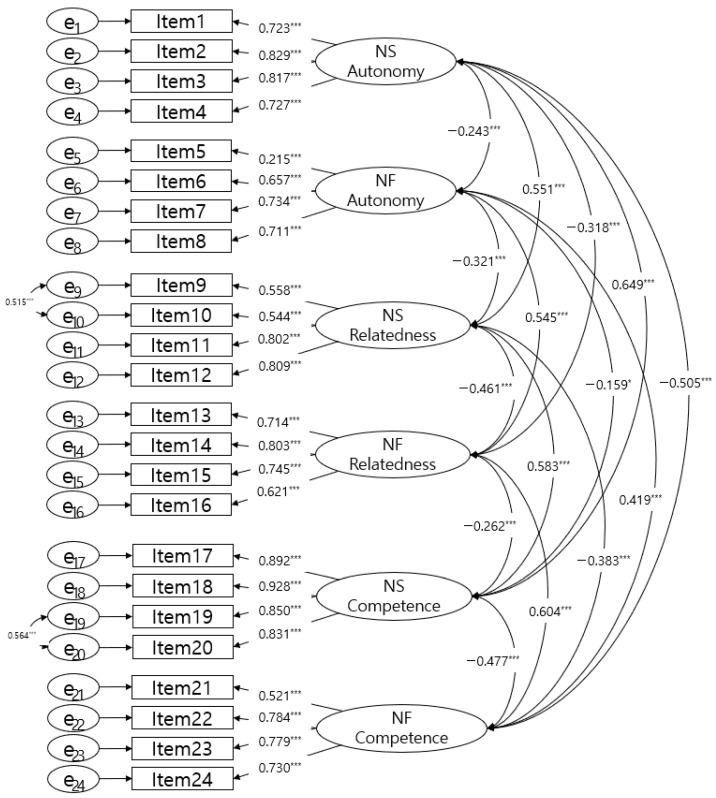
Six-dimensional structure with standardized parameter estimates: Results from the CFA of the K-BPNSFS. Note. K-BPNSFS: Korean version of the Basic Psychological Needs Satisfaction and Frustration Scale; NS: needs satisfaction; NF: needs frustration; CFA: Confirmatory Factor Analysis; chi-square test of model fit = 447.334 (df = 235), Root Mean Square Error of Approximation (RMSEA) = 0.050 (0.043, 0.057); Comparative Fit Index (CFI) = 0.953; Tucker–Lewis Index (TLI) = 0.945, * *p* < 0.05, *** *p* < 0.001.

The goodness of fit of the six-factor structural model of the K-BPNSFS was as follows: χ^2^ = 447.334 (df = 235, *p* < 0.001); RMSEA = 0.050 (90% CI: 0.043 to 0.057); CFI = 0.953; TLI = 0.945; SRMR = 0.059. Item 5 had a factor loading of 0.215, which falls below the typical threshold for acceptability in confirmatory factor analysis. However, it was retained due to its strong correlations with other items and its significant contribution to the structural model. This decision was further supported by the overall favorable fit indices and patterns observed in previously translated versions [20,23], which also indicated comparatively low factor loading for this item. Table 1 presents the goodness-of-fit indices for the model, and Figure 1 provides a detailed depiction of the six-factor structure of the K-BPNSFS. These results affirm the robustness and adequacy of the six-factor structure, aligning with the original BPNSFS, in accordance with established guidelines [43,44].

Additionally, CFA conducted on two specific subgroups by cancer type reaffirmed the consistency of the six-factor structure with the original scale. For the breast-cancer subgroup, the fit indices were as follows: χ^2^ = 405.48 (df = 235, *p* < 0.001); RMSEA = 0.057 (90% CI: 0.048 to 0.067); CFI = 0.940; TLI = 0.942; SRMR = 0.070. For the blood-cancer subgroup, the fit indices were as follows: χ^2^ = 323.04 (df = 235, *p* < 0.001); RMSEA = 0.050 (90% CI: 0.036 to 0.063); CFI = 0.950; TLI = 0.941; SRMR = 0.064. These findings underscore the scale’s robust applicability and validity across different cancer survivor populations.

### 3.3. Criterion-Related Convergent Validity

Table 2 presents correlations between the six dimensions of the K-BPNSFS and theoretically relevant constructs, supporting the criterion-related convergent validity. The dimension of autonomy demonstrated significant correlations with self-acceptance and depressive symptoms: Satisfaction with the need for autonomy was positively associated with self-acceptance (r = 0.588, *p* < 0.001) and negatively with depressive symptoms (r = −0.390, *p* < 0.001). In contrast, frustration in the need for autonomy showed negative correlations with self-acceptance (r = −0.272, *p* < 0.001) and positive correlations with depressive symptoms (r = 0.178, *p* < 0.001).

The dimensions related to the need for relatedness showed significant associations with family interaction. Satisfaction with the need for relatedness was positively correlated with family interaction (breast-cancer: r = 0.502, *p* < 0.001; blood-cancer: r = 0.369, *p* < 0.001). In comparison, frustration in the need for relatedness was negatively correlated with family interaction (breast-cancer: r = −0.404, *p* < 0.001; blood-cancer: r = −0.300, *p* < 0.001).

Regarding the need for competence, satisfaction was positively correlated with self-efficacy (r = 0.579, *p* < 0.001) and negatively correlated with uncertainty (r = −0.296, *p* < 0.001). Conversely, frustration in the need for competence was negatively associated with self-efficacy (r = −0.542, *p* < 0.001) and positively associated with uncertainty (r = 0.396, *p* < 0.001).

### 3.4. Reliability

The internal consistency of each of the six dimensions of the K-BPNSFS was evaluated using Cronbach’s alpha coefficients, inter-item correlations, and item-total correlations.

Table 1 and Table 3 detail the internal consistency metrics for the sub-dimensions of the K-BPNSFS. The alpha coefficient for need satisfaction for autonomy was 0.851, indicating good reliability. The reliability for need frustration for autonomy was slightly lower at 0.672, suggesting moderate internal consistency. Need satisfaction for relatedness showed a Cronbach’s alpha of 0.810, while need frustration for relatedness had an alpha of 0.803, indicating good reliability. The satisfaction of competence needs demonstrated excellent internal consistency, with a Cronbach’s alpha of 0.938. The reliability for frustration of competence needs was also acceptable, with a Cronbach’s alpha of 0.793. Table 3 presents the Cronbach’s alpha values for each item if deleted, as derived from the item analysis.

The inter-item correlations (0.238 ≤ r ≤ 0.872) within the K-BPNSFS indicated significant internal consistency among the items. The results met the acceptable range criteria, exceeding 0.15. Additionally, except for item 5, the item-total correlations (0.455 ≤ r ≤ 0.873), which measure how well each item correlates with the total score, were within an adequate range. These results indicate that the items are related but not redundant, which is desirable on a psychometric scale.

## 4. Discussion

As an inherent aspect of human nature, BPN play a key role in activating intrinsic motivation and behavioral outcomes intricately associated with personal growth and psychological well-being [8,9]. This study validated the reliability and applicability of the K-BPNSFS for assessing the satisfaction and frustration of BPN—autonomy, relatedness, and competence—among cancer survivors in Korea.

Our findings affirm that, consistent with the original version, the K-BPNSFS consists of a six-dimensional structure classified into satisfaction and frustration dimensions for each BPN. This structure also aligns with the features verified in other translated versions across diverse populations from Western and Asian countries [20,21,22,23]. While BPN are considered an intrinsic trait of human beings, and the BPNSFS has been validated for assessing BPN in individuals with specific health-related issues [23,24], its applicability, specifically among cancer survivors, has been underexplored. Therefore, our findings significantly contribute by demonstrating that the K-BPNSFS is a reliable, valid, and culturally appropriate tool for the oncological context in Korea. Furthermore, these findings support the foundational principles of SDT, emphasizing the universality of BPN across various populations and socio-cultural environments [10,11,12,16].

The current study confirmed the convergent validity of the K-BPNSFS by demonstrating that satisfaction and frustration in each of the BPN are associated with psycho-cognitive constructs in directions that are both theoretically and empirically supported [3,4,5,6,7,8]. Specifically, higher levels of satisfaction in needs for autonomy, relatedness, and competence were associated with greater self-acceptance, family interaction, and self-efficacy, respectively. Conversely, higher levels of frustration in needs for autonomy, relatedness, and competence were linked to more depressive symptoms, poorer family interaction, and increased uncertainty. These results align with existing theoretical and empirical evidence, which indicates that distinct values between satisfaction and frustration for specific needs are inversely related to well-being and ill-being [30,35,38,39,49]. Therefore, the evidence of convergent validity underscores the value of the K-BPNSFS as an effective instrument for assessing the unique status of BPN among cancer survivors, highlighting its critical role in developing strategies to enhance self-directed motivation and overall well-being.

This study demonstrated strong internal consistency across the six dimensions of the K-BPNSFS. Acceptable inter-item and item-total correlations demonstrate robust internal consistency, suggesting that the items are well-aligned with the overall measured construct. Cronbach’s alpha coefficients of the satisfaction sub-dimensions were 0.851 for autonomy, 0.810 for relatedness, and 0.938 for competence, all exceeding the acceptable threshold of 0.7 [36,47]. For the frustration sub-dimensions, alpha values were 0.672 for autonomy, 0.803 for relatedness, and 0.793 for competence, showing acceptable but slightly lower reliability than the satisfaction sub-dimensions. The relatively lower internal consistency in the frustration of the autonomy needs may reflect the complex and multifaceted nature of this frustration, which encompasses feelings of constraint and lack of control [50]. This finding may also be influenced by cultural differences, as autonomy is often less emphasized and perceived as a less inherent resource in Eastern cultural contexts compared to Western cultures [51,52]. Furthermore, Cronbach’s alpha can underestimate reliability for scales with diverse item content or a small number of items [53], as is the case with this four-item sub-dimension. Notably, the acceptable item-total correlations for the autonomy frustration dimension indicate that each item contributes significantly to the overall measurement, supporting the scale’s validity. Therefore, the slightly lower alpha value does not detract from the scale’s construct validity.

While this study provides valuable insights, its findings have limited generalizability due to the focus on two independent samples of breast- and blood-cancer survivors. Additionally, the sample consisted of relatively stable cancer survivors, excluding individuals with advanced or terminal-stage cancer. Future research should address to overcome these limitations by incorporating a more diverse range of cancer types and stages as well as larger sample sizes to enhance the robustness and applicability of the findings across broader populations. Moreover, while the existing literature highlights the relevance of BPN across different age groups, evidence from extensive cohort studies suggests that the fulfillment and frustration of BPN can vary depending on life events and circumstances [54]. Additionally, the participants in this study were predominantly middle-aged to older adults, which aligns with the typical age range for cancer incidence. Although previous research has confirmed the six-dimensional structure of the BPNSFS in diverse age groups, including adolescents and young adults [55], further studies involving a broader age range are necessary to fully establish the scale’s generalizability across the lifespan. Therefore, additional research is needed to strengthen the evidence base and validate the applicability of the K-BPNSFS across diverse populations.

The SDT framework has been applied to psycho-cognitive behavioral studies to understand the unique psychological needs of individuals faced with diverse challenges through socio-cultural living contexts and their links with internal motivation, self-driven behaviors, and health outcomes [7,8]. Our findings substantiate that the K-BPNSFS can capture the psychological needs of cancer survivors in the distinguished aspects of satisfaction and frustration of BPN. As such, the validated application of this scale shows promise in guiding cancer survivors toward self-regulated motivation and informed decision making related to behavioral changes, ultimately enhancing their overall quality of life. Furthermore, our findings broaden its applicability and support cross-cultural comparisons, contributing to a more comprehensive understanding of the psychological needs of individuals with chronic health concerns, including cancer survivors.

## 5. Conclusions

Understanding fundamental psychological needs is essential for fostering intrinsic motivation, which drives self-directed behaviors and enhances well-being among cancer survivors. This study establishes that the K-BPNSFS is a valid, reliable, and culturally appropriate tool for assessing the satisfaction and frustration of each BPN in a cohort of Korean cancer survivors.

Utilizing the K-BPNSFS enables a comprehensive assessment of the unique psychological needs of cancer survivors, marking a crucial step in promoting intrinsic motivation and self-determined behaviors that contribute to psychological well-being. To further confirm the K-BPNSFS as an essential tool for clinical guidance, additional studies are recommended involving cancer populations with varied cancer types and socio-demographic characteristics. Such studies would enhance the evidence base for the K-BPNSFS’s applicability, thereby providing valuable insights that could inform clinical practices for fostering intrinsic motivation and self-driven behaviors throughout the cancer journey.

## Figures and Tables

**Table 1:** Construct validity and reliability of the K-BPNSFS (N = 367).

Sub-Dimensions		Item	FL	EE	AVE	CR	Cronbach’s α
Autonomy	Satisfaction	Item 1	0.723	0.030			
		Item 2	0.829	0.022	0.601	0.989	0.851
		Item 3	0.817	0.023			
		Item 4	0.727	0.030			
	Frustration	Item 5	0.215	0.058			
		Item 6	0.657	0.042	0.381	0.990	0.672
		Item 7	0.734	0.038			
		Item 8	0.711	0.039			
Relatedness	Satisfaction	Item 9	0.558	0.043			
		Item 10	0.544	0.044	0.476	0.994	0.810
		Item 11	0.802	0.030			
		Item 12	0.809	0.030			
	Frustration	Item 13	0.714	0.032			
		Item 14	0.803	0.026	0.524	0.995	0.803
		Item 15	0.745	0.030			
		Item 16	0.621	0.038			
Competence	Satisfaction	Item 17	0.892	0.014			
		Item 18	0.928	0.011	0.767	0.997	0.938
		Item 19	0.850	0.017			
		Item 20	0.831	0.019			
	Frustration	Item 21	0.521	0.043			
		Item 22	0.784	0.028	0.506	0.998	0.793
		Item 23	0.779	0.028			
		Item 24	0.730	0.031			

Note. K-BPNSFS: Korean version of the Basic Psychological Needs Satisfaction and Frustration Scale; AVE: average variance extracted; CR: composite reliability; EE: error estimate; FL: factor loading.

**Table 2:** Correlations between six dimensions of K-BPNSFS and theoretically relevant construct.

	Autonomy		Relatedness		Competence	
	Satisfaction r (*p*)	Frustration r (*p*)	Satisfaction r (*p*)	Frustration r (*p*)	Satisfaction r (*p*)	Frustration r (*p*)
Self-acceptance ^1^	0.588 (<0.001)	−0.272 (<0.001)	-	-	-	-
Depression ^2^	−0.390 (<0.001)	0.178 (<0.001)	-	-	-	-
Family interaction ^1^	-	-	0.502(<0.001)	−0.404 (<0.001)	-	-
Family interaction ^2^	-	-	0.369 (<0.001)	−0.300 (<0.001)	-	-
Self-efficacy ^1^	-	-	-	-	0.579 (<0.001)	−0.542 (<0.001)
Uncertainty ^2^	-	-	-	-	−0.296 (<0.001)	0.396 (<0.001)

Note. K-BPNSFS: Korean version of the Basic Psychological Needs Satisfaction and Frustration Scale. ^1^ Breast-cancer survivors (n = 220); ^2^ blood-cancer survivors (n = 147).

**Table 3:** K-BPNSFS item analysis: Means, standard deviations, and Cronbach’s alpha if item deleted (N = 367).

		Item	M (SD)	ITC r	Cronbach’s α If Item Deleted
Satisfaction of autonomy needs					
	1	I feel a sense of choice and freedom in the things I undertake	4.136 (1.080)	0.647	0.830
	2	I feel that my decisions reflect what I really want	4.071 (0.986)	0.740	0.792
	3	I feel my choices express who I really am	3.937 (1.002)	0.739	0.791
	4	I feel I have been doing what really interests me	3.823 (1.123)	0.649	0.831
Frustration of autonomy needs					
	5	Most of the things I do feel like “I have to”	3.657 (1.170)	0.213	0.735
	6	I feel forced to do many things I wouldn’t choose to do	3.864 (1.026)	0.468	0.602
	7	I feel pressured to do too many things	4.174 (0.982)	0.607	0.488
	8	My daily activities feel like a chain of obligations	4.362 (0.870)	0.558	0.528
Satisfaction of relatedness needs					
	9	I feel that the people I care about also care about me	3.855 (1.064)	0.648	0.757
	10	I feel connected with people who care for me, and for whom I care	3.918 (1.068)	0.651	0.750
	11	I feel close and connected with other people who are important to me	3.785 (1.099)	0.660	0.747
	12	I experience a warm feeling with the people I spend time with	3.785 (1.086)	0.571	0.789
Frustration of relatedness needs					
	13	I feel excluded from the group I want to belong to	3.943 (1.037)	0.629	0.750
	14	I feel that people who are important to me are cold and distant towards me	1.839 (1.065)	0.677	0.729
	15	I have the impression that people I spend time with dislike me	2.481 (1.386)	0.636	0.749
	16	I feel the relationships I have are just superficial	2.297 (1.356)	0.559	0.787
Satisfaction of competence needs					
	17	I feel confident that I can do things well	1.733 (1.068)	0.823	0.928
	18	I feel capable at what I do	1.462 (0.865)	0.861	0.916
	19	I feel competent to achieve my goals	1.403 (0.840)	0.873	0.912
	20	I feel I can successfully complete difficult tasks	1.883 (1.061)	0.851	0.919
Frustration of competence needs					
	21	I have serious doubts about whether I can do things well	2.500 (1.202)	0.455	0.812
	22	I feel disappointed with many of my performance	2.144 (1.180)	0.684	0.701
	23	I feel insecure about my abilities	2.234 (1.178)	0.665	0.710
	24	I feel like a failure because of the mistakes I make	1.956 (1.230)	0.618	0.734

Note. K-BPNSFS: Korean version of the Basic Psychological Needs Satisfaction and Frustration Scale; ITC: item-total correlation.

## Data Availability

The data supporting this study’s findings are not publicly available since participants did not give written consent for their data to be shared publicly.
